# Monitor unit optimization in RapidArc plans for prostate cancer

**DOI:** 10.1120/jacmp.v14i3.4114

**Published:** 2013-05-06

**Authors:** Stefania Clemente, Mariella Cozzolino, Costanza Chiumento, Alba Fiorentino, Rocchina Caivano, Vincenzo Fusco

**Affiliations:** ^1^ Medical Physics IRCCS CROB Rionero in Vulture PZ Italy; ^2^ Department of Radiation Oncology IRCCS CROB Rionero in Vulture PZ Italy

**Keywords:** RapidArc prostate, MU optimization tool, integral dose, gamma index

## Abstract

Intensity‐modulated radiation therapy (IMRT) has become a standard treatment for prostate cancer based on the superior sparing of the bladder, rectum, and other surrounding normal tissues compared to three‐dimensional conformal radiotherapy, despite the longer delivery time and the increased number of monitor units (MU). The novel RapidArc technique represents a further step forward because of the lower number of MUs per fraction and the shorter delivery time, compared to IMRT. This paper refers to MU optimization in RA plans for prostate cancer, using a tool incorporated in Varian TPS Eclipse. The goal was to get the lowest MU RA plan for each patient, keeping a well‐defined level of PTV coverage and OAR sparing. Seven prostate RA plans (RA MU‐Optimized) were retrospectively generated using the MU optimization tool in Varian Eclipse TPS. Dosimetric outcome and nontarget tissue sparing were compared to those of RA clinical plans (RA Clinical) used to treat patients. Compared to RA Clinical, RA MU‐Optimized plans resulted in an about 28% (p=0.018) reduction in MU. The total integral dose (ID) to each nontarget tissue (but not the penile bulb) showed a consistent average relative reduction, statistically significant only for the femoral heads. Within the intermediate dose region (40–60 Gy), ID reductions (4%−17% p<0.05) were found for the rectum, while a slight but significant (0.4%−0.9%,p<0.05) higher ID was found for the whole body. Among the remaining data, the mean dose to the bladder was also reduced (−12%,p=0.028). Plans using MU optimization are clinically applicable and more MU efficient, ameliorating the exposure of the rectum and the bladder to intermediate doses.

PACS number: 87

## I. INTRODUCTION

The development of intensity‐modulated radiation therapy (IMRT) has enabled the delivery of highly conformal dose distributions to the target along with higher sparing of critical normal tissues, which becomes important in sites where tumors are in close proximity or abutting critical normal structures.^(1)^ For prostate cancer treatment, IMRT has become an optimal technique, given the geometric relationships of the target volume to the bladder, the rectum, and the surrounding normal tissue.^(2)^ The dosimetric advantages of both an increased conformity and a greater normal tissue sparing with IMRT compared to three‐dimensional conformal radiotherapy (3D CRT) allowed safer dose escalation, as the dose to adjacent critical structures can be more easily maintained below tolerance.^(3)^

However, the potential downsides of IMRT include the longer time required for treatment delivery and the higher number of monitor units (MU) per plan, resulting in a larger total body radiation dose because of radiation leakage and internal scatter. It has been estimated that MU demand doubled for IMRT compared to 3D CRT, with a potential increase in the risk of secondary cancers by a factor of 1.2–8.^4^, ^5^

RapidArc (RA) and volumetric‐modulated arc therapy (VMAT) techniques represent attractive solutions because of the lower number of MUs per fraction and shorter delivery time compared to dynamic sliding window IMRT.^6^, ^7^ Palma et al.^(6)^ showed that RA achieved a 42% relative decrease in the mean number of MUs required for delivery treatment in prostate cancer over IMRT. Yoo et al.^(8)^ found that, for PTVs including the prostate and the seminal vesicles, the average values of total MUs in IMRT were 42% and 37% greater than those in one‐arc and two‐arc RA plans, respectively, and that the delivery required approximately 3.4 or 1.8 min less than IMRT, respectively.

The present paper refers to MU optimization in RA plans for prostate cancer. The goal was to get the lowest MU RA plan for each patient selected for the study, using the “MU optimization” tool incorporated in Varian Treatment Planning System (TPS) Eclipse (Version 8.6), keeping a well‐defined level of PTV coverage and OAR sparing. Dosimetric outcome was evaluated in terms of nontarget tissue sparing and delivery efficiency, using different parameters such as integral dose (ID), delivery time, and gamma index.

## II. MATERIALS AND METHODS

### A. Patient selection and contouring

We randomly selected seven patients treated for prostate cancer at our institution with the RapidArc technique in 2011. For all patients, plans were run on CT scans acquired with 5 mm slice thickness in the supine position. Patients were instructed to be scanned and treated with a full bladder and an empty rectum according to in‐house guidelines. The clinical target volume (CTV) and organs at risk (OAR) were delineated according to Radiation Therapy Oncology Group (RTOG) guidelines.^(9)^ CTV included the prostate and the proximal (1.5 cm) seminal vesicles. The PTV was generated by adding a 0.8 cm margin to the CTV in all directions, except craniocaudally where a 1 cm margin was used. The relevant OAR structures were the rectum, the bladder, the femoral heads, the penile bulb, and the small bowel. The rectum was contoured, from the first CT slice below the sigmoid flexure to its caudal limit, defined as the first CT slice above anal verge.^(10)^ Both the rectum and the bladder contours included filling. For the bowel, all loops were contoured up to 2 cm above the superior extent of PTV.

### B. Planning and rules

RapidArc plans were generated following specific planning rules, using Eclipse TPS (Version 8.6; Varian Medical Systems, Palo Alto, CA) for 6 MV photons beams in a Varian Trilogy machine with 120‐leaf millennium MLC. The Anisotropic Analytical Algorithm (AAA) dose calculation algorithm was used. The progressive resolution optimizer (PRO) was used to optimize RA plans as described elsewhere.^11^, ^12^ This optimization algorithm is used to determine the combination of field shapes and segment weights (with dose rate and gantry speed variations) which best approximate the desired dose distribution in the inverse planning problem. The total prescription dose was 80 Gy to PTV at a daily dose of 2 Gy. RA plans included one arc field rotating counterclockwise from 179.9° to 180.1°, with 2° control point spacing, the collimator rotated to 45°, and a dose rate of 600 MU/min as upper limit. The dose/volume objectives for PTV and OAR are reported in Table 1. Plans aimed at achieving PTV coverage (95% of each PTV covered by at least 95% of the prescribed dose, D95%≥95%) without violating OAR sparing (rectum, bladder, femoral heads, penile bulb) and hotspots (D2%).

**Table 1:** Dose‐volume constraints and average (± SD) dosimetric results for planning target volume and OARs.

*Structures*	*Dose Index*	*Objectives*	*RA Clinical*	*RA MU‐Optimized*	*p*
PTV	$D_{mean}(\%)$	100	100.7±1.0	100.6±1.0	0.345
	D2% (%)	≤107	103.8±1.4	104.1±1.4	0.310
	D95% (%)	≥95	96.4±0.5	95.6±0.5	0.210
	HI	—	5.6±0.8	6.9±1.3	0.018^a^
	CI	—	0.95±0.01	0.95±0.01	0.344
Bladder	V60 (%)	<35	32.5±3.7	32.1±2.7	0.344
Rectum	V50 (%)	<50−55	45.7±4.9	51.1±3.5	0.018^a^
	V60 (%)	<40−45	31.6±3.6	34.8±3.3	0.026^a^
	V70 (%)	<25	20.4±2.6	21.7±3.4	0.593
Penile Bulb	$D_{mean}\;(Gy)$	50	47.2±5.1	47.5±6.6	0.916
Femoral Heads	$D_{mean}\;(Gy)$	45	27.0±2.0	29.6±0.4	0.053
Small Bowel	$D_{\max}\;(Gy)$	55	5.9±3.7	6.0±3.6	0.496

^a^ Differences statistically significant (p<0.05).

PTV = planning target volume; CI = conformity index; HI= homogeneity index.

For each patient a plan was generated for treatment (RA Clinical plan). This is considered here the reference plan, without further optimization. As part of the study, a second RA plan was also run using the same objectives and rules of clinical plans, but adding constraints on MU (RA MU‐Optimized) as described below.

### C. Mu tool in the progressive resolution optimizer (PRO)

For RA MU‐Optimized plans, a MU optimization tool that is available in PRO was used. This allows increasing or decreasing manually the modulation of the plan acting on MU numbers. In this tool, three parameters need to be set: strength (S), maximum (Max MU) and minimum (Min MU) MU number. The S parameter forces the optimizer to reach the MU goal within the established Min and Max MU boundaries.

Due to the lack of literature, preliminary planning exercises were run to investigate the effects of S, Min, and Max variation on PTV coverage. The S parameter, following manufacturer directions, was set to 50 and 100, while the Min and Max MU parameters varied between 75% and 25% of MU number obtained in the RA Clinical plan.

### D. Plan comparison

#### D.1 Plan quality

To assess the compliance with the dosimetric rules and objectives assigned, an analysis based on cumulative dose‐volume histograms (DVH) was performed for each patient and plan. All plans (RA Clinical and RA MU‐Optimized) were normalized to keep the same mean dose to the PTV. Metrics to assess plans’ quality with respect to the PTV coverage and OARs sparing are reported in Table 1. In the table, the homogeneity index (HI) was defined as the difference between the percentage dose covering 5% and 95% of the PTV (HI=D5%−D95%); and the conformity index (CI) was defined as the ratio between the volume of tissue receiving 95% of the PTV prescribed dose and the volume of PTV. Lower values of HI and CI represent better PTV homogeneity and conformity, respectively.

#### D.2 Delivery efficiency: MU and treatment time

MUs and delivery times were analyzed for both techniques. The delivery time was manually measured as the time from beam on to beam off.

#### D.3 Healthy tissue sparing: integral dose

The integral dose (ID) was analyzed to evaluate the sparing of nontarget tissues, defined as the OARs minus the target. It was calculated using the equation:

(1) $$\text{ID}=\Sigma_{i}\times D_{i}\times V_{i}\times P_{i}^{\left(13\right)}$$

where $V_{i}$ is the volume of the nontarget tissue irradiated at a dose of $D_{i}$,and $P_{i}$ is the local density of $V_{i}$. The current definition of ID differs from the conventional one^(14)^ and describes the total energy delivered to a specific normal tissue — in this case, to the bladder, the rectum, the penile bulb, the femoral heads, the small bowel, and the whole body.

The total ID includes the volume of tissue receiving all dose levels; the dose region ID investigates the amount of tissue receiving a maximum dose of interest (i.e., up to 10 Gy, up to 20 Gy). IDs were determined from DVH data by using the DVH differential function of the planning software at bins of 1 cGy and assuming a unit density for the pelvis. Further analysis of nontarget tissues was performed using mean ($D_{\text{mean}}$) and maximum doses ($D_{\text{max}}$).

#### D.4 Plan delivery: gamma evaluation

The doses delivered were measured with a commercial 2D array ionization chamber (MatriXX, IBA Dosimetry, Schwrazenbruck, Germany) equipped with 729 ionization chambers uniformly arranged in a 27×27 matrix with an active area of $27\times 27\;{cm}^{2}$. A cubic‐shaped phantom (MultiCube Phantom, IBA Dosimetry) with a suitable cavity is used to insert the 2D ion chamber array. Details of the design, accuracy, and use of the MatriXX MultiCube phantom have previously been discussed.^(15)^ The gamma (γ) evaluation method proposed by Low et al.^(16)^ was used to quantify the results. Reference gamma index value was set at 3% dose agreement (DA) within 3 mm distance to agreement (DTA).

### E. Statistical analysis

Plan comparisons were done with the Wilcoxon matched‐pair signed‐rank test for nonparametrically distributed data. The threshold for statistical significance was set at p<0.05. All statistical analysis was performed using SPSS Version 17.0 (SPSS Inc., Chicago, IL).

## III. RESULTS

### A. Parameter setting for the MU optimizer tool

Results of the preliminary study to set S, Max, and Min MU parameters in the optimization tool are reported in Table 2. The percent variation of both the PTV homogeneity index (HI) and the number of MU in the RA MU‐Optimized plans with respect to the corresponding RA clinical plans were evaluated. The best combination is reached when the decrease in MU is maximal while the deterioration in HI is minimal, weighting more the former over the latter. All seven patients were considered. According to Table 2, the most favorable combination is reached when S=100,Max MU=50%, and Min MU=0%. Therefore, these values were set for the optimization parameters.

**Table 2:** Homogeneity index (HI) and monitor unit number (MU) as a function of S, Min, and Max MU parameters. Results are reported as percent variation for RA MU‐Optimized plans over RA Clinical ones (seven patients).

*MU Parameters*			*Diff %*	
*S*	*Min MU*	*Max MU*	*mean* Δ*HI*	*mean* Δ*MU*
50	0%	75%	−3.5%	+2.7%
		50%	−35.6%	−7.3%
		25%	−1.8%	+3.2%
100	0%	75%	−41.5%	−20.4%
		50%	−23.0%	−28.0%
		25%	−21.0%	−16.5%
50	50%	75%	−6.5%	+2.5%
		50%		
100	50%	75%	−59.8%	−20.4%
		50%		

S=Strength; Min/Max MU=minimum and maximum MU number; Diff% (relative difference in %); ΔHI=(HI RA MU−Optimized−HI RA Clinical)/HI RA Clinical×100; ΔMU=(MU RA MU−Optimized−MU RA Clinical)/MU RA Clinical×100.

### B. Plan quality

**Figure 1:**
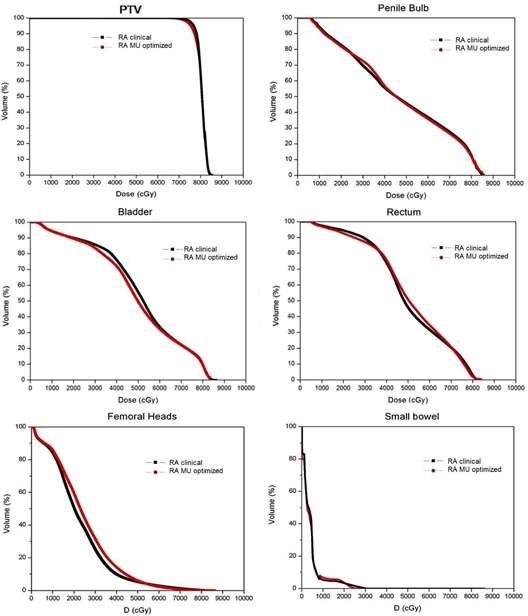
Average dose–volume histograms for seven clinical RA Clinical plans (black line) and RA MU‐Optimized plans (red line) for PTV and OARs: penile bulb, bladder, rectum, femoral heads and small bowel.

**Figure 2:**
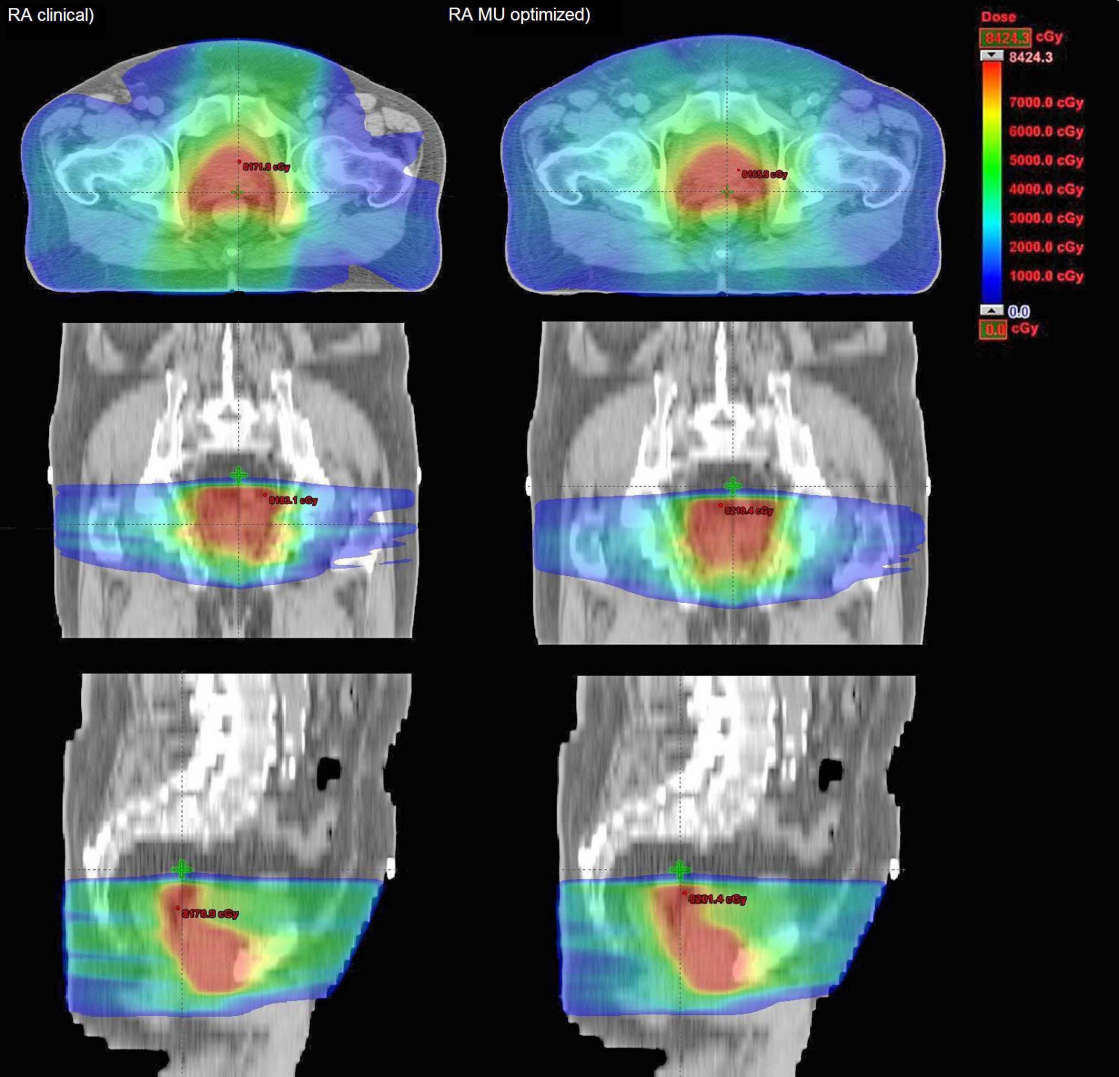
Isodose distributions on axial, frontal, and sagittal views for one representative case for both RA Clinical and MU‐Optimized plans.

Table 1 summarizes the results for PTV and OARs by each plan modality. The average cumulative DVH (seven‐patient average, Fig. 1) shows the general dosimetric trends of PTV and OARs for RA Clinical and RA MU Optimized plans, while Table 3 reports numerical results. Histograms are built by averaging the corresponding volumes over the whole patient's cohort at each dose bin (1 cGy in this case). Figure 2 shows isodose distributions on axial, frontal, and sagittal views for one representative case of both RA Clinical and RA MU‐Optimized plans.

RA Clinical plans provided a slightly superior PTV coverage compared to RA MU‐Optimized plans; however, a clinically acceptable level of coverage has been always reached with either plan. On average, a slightly worse dose homogeneity was observed for RA MU‐Optimized over RA Clinical plans (−23%,p<0.018). However, the conformity index was similar among the plans (see Table 1). Both strategies met the dose objectives for all OARs (bladder, rectum, penile bulb, femoral heads, small bowel) with a slight but statistically significant difference in favor of RA Clinical over RA MU‐Optimized plans for the rectum (Table 1).

**Table 3:** MU number and delivery times, obtained by RA Clinical plans and RA MU‐Optimized plans.

	*RA Clinical*	*RA MU‐Optimized*	
*Patient*	*MU number*	*p*
1	785	537	
2	656	515	
3	576	399	
4	527	401	
5	492	360	
6	577	413	
7	774	556	
mean	627±107	454±73	0.018^a^
*Delivery time (min)*			
mean	1.34±0.06	1.28±0.02	0.090

^a^ Differences statistically significant (p<0.05).

### C. Delivery efficiency: MU and treatment time

For both RA Clinical and RA MU‐Optimized plans, MU number and delivery time are shown in Table 3. The use of MU optimization reduced the MU number by an average of 28% (627 vs. 454 per fraction p=0.018). Delivery times (Table 3) were not statistically different for both optimization methods (‐4% for RA MU‐Optimized, p=0.09).

### D. Healthy tissue sparing: integral dose

**Figure 3:**
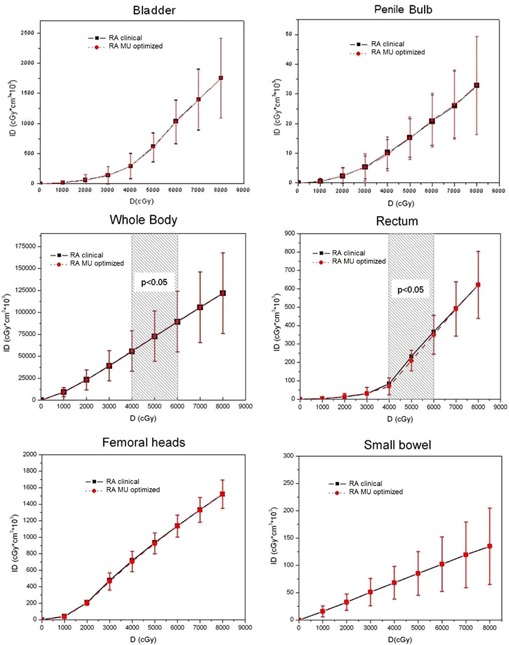
Mean total integral dose (ID) curves to nontarget tissues, for both RA Clinical (black line) and RA MU‐Optimized plans (dashed red line). The dashed areas highlight dose regions with statistically significant difference (p<0.05).

The total ID at all dose levels and $D_{\text{mean}}$ and $D_{\text{max}}$ to nontarget tissues are listed in Table 4. The average integral dose curves were plotted across all dose levels in Fig. 3.

Using MU optimization, the total ID to each nontarget tissue, except the penile bulb, showed a consistent average relative reduction that was statistically significant only for the femoral heads (Table 4). Within the intermediate dose region (40–60 Gy), statistically significant reductions (4%–17%) were found for the rectum (Table 5). Conversely, a slight (0.4%–0.9%) but significant higher ID to the whole body was found (Table 5).

Among the remaining data (Table 4), the $D_{\text{mean}}$ to the bladder was reduced with RA MU‐Optimized over RA Clinical plans. IDs to penile bulb, femoral heads, and small bowel were similar at all dose levels.

**Table 4:** Total integral dose (ID), $D_{\text{mean}}$, and $D_{\text{max}}$ to nontarget tissues.

*Nontarget Tissues*	*Index*	*RA Clinical*	*RA MU‐Optimized*	*Diff %*	*p*
Bladder	$D_{mean}\;(Gy)$	51.4±3.3	45.0±10.0	−12.4	0.028^a^
	$D_{\max}\;(Gy)$	84.3±1.3	78.2±15.5	−7.2	0.279
	$ID(Gy\times m^{3}\times 10^{3})$	53.3±21.7	52.9±21.4	−0.7	0.064
Rectum	$D_{mean}\;(Gy)$	51.0±3.0	51.4±2.5	+0.8	0.068
	$D_{\max}\;(Gy)$	83.0±1.4	82.1±1.2	−1.1	0.059
	$ID(Gy\times m^{3}\times 10^{3})$)	18.4±5.4	17.8±5.4	−3.2	0.176
Penile bulb	$D_{mean}\;(Gy)$	47.2±5.1	47.5±6.6	+0.6	0.916
	$D_{\max}\;(Gy)$	82.9±0.9	83.3±1.3	+0.5	0.070
	$ID(Gy\times m^{3}\times 10^{3})$)	1.1±0.5	1.1±0.6	0.0	0.735
Femoral heads	$D_{mean}\;(Gy)$	27.0±2.0	29.6±0.4	+9.6	0.053
	$D_{\max}\;(Gy)$	85.0±0.8	86.0±1.6	+1.1	0.743
	$ID(Gy\times m^{3}\times 10^{3})$)	63.4±30.5	63.0±30.0	−0.6	0.028^a^
Small bowel	$D_{mean}\;(Gy)$	3.8±2.5	3.7±2.4	−2.6	0.416
	$D_{\max}\;(Gy)$	31.0±3.7	30.2±3.6	+1.7	0.496
	$ID(Gy\times m^{3}\times 10^{3})$)	13.7±4.5	13.6±4.4	−0.7	0.317
Whole Body	$ID(Gy\times m^{3}\times 10^{3})$)	5169.3±2048.6	5182.3±2061.3	+0.3	0.141

^a^ Differences statistically significant (p<0.05).

Diff% (relative difference in %) = (RA MU‐Optimized‐RA Clinical)/RA Clinicalx100; ID = total integral dose at all dose level.

**Table 5:** Integral dose for the rectum and for the whole body (nontarget tissue) in the low‐ to high‐dose region.

	${ID}_{Rectum}(Gy\times {cm}^{3}\times 10^{3})$				${ID}_{WB}(Gy\times {cm}^{3}\times 10^{3})$			
*Dose Region (Gy)*	*(RA Clinical)*	*(RAMU‐Optimized)*	*P*	*Diff %*	*(RA Clinical)*	*(RA MU‐Optimized)*	*p*	*Diff*%
10	0.031	0.032	0.461	+3.2	93.3	91.5	0.128	−1.9
20	0.125	0.135	0.225	+8.0	233.3	231.1	0.398	−0.9
30	0.297	0.311	0.463	+4.7	390.8	393.3	0.397	+0.6
40	0.843	0.700	0.040^a^	−17	556.1	561.1	0.048^a^	+0.9
50	2.324	2.097	0.018^a^	−9.7	725.5	730.3	0.028^a^	+0.7
60	3.665	3.504	0.028^a^	−4.4	893.0	896.2	0.027^a^	+0.4
70	4.928	4.903	0.352	−0.5	1057.5	1059.0	0.066	+0.1
80	6.214	6.214	0.128	0.0	1220.0	1220.0	0.128	0.0

^a^ Differences statistically significant (p<0.05).

IDRectum = integral dose to rectum and IDWB = integral dose to whole body in the low‐to high‐dose region for RA clinical plans and RA MU‐Optimized; Diff% (relative difference in %) = (RA MU‐Optimized ‐ RA Clinical)/RA Clinical × 100.

### E. Plan delivery: gamma evaluation

At 5% threshold criteria, the average passing rate was 98.0±3.0% for RA Clinical and 98.5±3.2% for RA MU‐Optimized plans. The relative difference was small (0.5%) and not statistically significant (p=0.498).

## IV. DISCUSSION

The incidence of second malignancies (SMs) for prostate cancer patients treated with radiotherapy remains unclear. After conformal RT (3D CRT), 15% and 34% of patients have been reported to develop SMs at five and ten years, respectively, mainly in organs in‐field such as the bladder and the rectum.^(17)^ Other studies suggested lower rates.^18^, ^19^, ^20^

While awaiting for a more solid and widespread clinical experience with longer follow‐up to assess the incidence of SM risks using modern techniques (also for RA/VMAT), the attempt to reduce MUs in daily practice seems justified given the potential association between the number of delivered MUs (due to leakage and scattered dose) and SMs.^4^, ^21^

Several studies have shown that the new volumetric techniques (RA/VMAT) reduce the number of MUs and delivery time compared to IMRT; the estimated reduction in MUs ranges from about 15% to 40%.^6^, ^8^, ^22^

Our study investigated the possibility of a further reduction of MUs, keeping a well‐defined level of both PTV coverage and OARs sparing. Using the MU optimization tool for RA prostate plans, we obtained a further average decrease of MU by about 28% compared to clinical RA plans. Therefore, we could estimate a delivered number of MUs that is less than half of the corresponding IMRT plan and twice of a typical conformal plan for prostate cancer.

The results obtained here are certainly related to the software used and specific to the anatomical region considered. The predetermined values for MU parameters, established during optimization planning exercises, strictly apply to the considered volumes, the specific geometry of beam configuration (one arc rather than two), as well as the planning objectives and priorities.

Besides the scattered dose and MUs, the volume of normal tissue within the low‐dose region (≤6 Gy)^(23)^ also seems to be correlated to the risk of SMs after radiotherapy. Moreover, tissues receiving higher doses (30–60 Gy) seems to be at higher risk of developing sarcomas.^(24)^ Whether IMRT would lead to an increase in integral dose (ID) over 3D CRT is controversial^2^, ^25^, ^26^, ^27^ With careful planning, Aoyama et al.^(28)^ were able to reduce the normal tissue ID by 5% with IMRT over 3D CRT for prostate cancer. Yang et al.^(29)^ also found a decrease in the ID to normal tissues and whole body (13% and 11%, respectively) in postoperative endometrial cancer patients with IMRT over 3D CRT. How RA/VMAT compares to IMRT in terms of ID is also unclear. Some studies on prostate treatment^30^, ^31^ reported comparable results for both techniques, while a study on cervical cancer^(32)^ found that ID was improved with RA over IMRT.

In our experience, the ID to the rectum was found to be reduced by up to 17% in the intermediate dose region, while the bladder mean dose was also significantly reduced. This could be due to a more rapid and symmetric falloff (higher selectivity) of dose distribution surrounding the target associated to RA MU‐Optimized over Clinical RA plans (Fig. 1). The practical implications of these reductions are difficult to be estimated since no validated model exists for calculating the risk in this region of intermediate–high doses.^(33)^ It has been pointed out that standard linear dose response models for secondary cancer induction may not apply due to cell killing or a balance between cell killing and repopulation.^(34)^

However, even if the clinical advantage cannot be quantified, it is always desirable to generate plans that lower the MU while not compromising target coverage, as we show here. The MU optimization tool is clinically applicable and ameliorates exposure for in‐field normal tissues (rectum and bladder), with lower internal scatter from patient and lower leakage from machine (using less MU number).

## V. CONCLUSIONS

Compared to the clinical RA plans, the use of a MU optimization tool produced plans with fewer MUs (on average −28%) while maintaining acceptable PTV coverage and OARs sparing. Additional studies need to evaluate MU optimization for other anatomic sites.
